# *Teucrium* Plant Species as Natural Sources of Novel Anticancer Compounds: Antiproliferative, Proapoptotic and Antioxidant Properties

**DOI:** 10.3390/ijms12074190

**Published:** 2011-06-27

**Authors:** Milan S. Stankovic, Milena G. Curcic, Jovana B. Zizic, Marina D. Topuzovic, Slavica R. Solujic, Snezana D. Markovic

**Affiliations:** 1 Department of Biology and Ecology, Faculty of Science, University of Kragujevac, Str. Radoja Domanovića No. 12, 34000 Kragujevac, Republic of Serbia; E-Mails: milenagen@gmail.com (M.G.C.); jovanazizic@gmail.com (J.B.Z.); marina@kg.ac.rs (M.D.T.); smarkovic@kg.ac.rs (S.D.M.); 2 Department of Chemistry, Faculty of Science, University of Kragujevac, Str. Radoja Domanovića No. 12, 34000 Kragujevac, Republic of Serbia; E-Mail: ssolujic@kg.ac.rs

**Keywords:** acridin orange/ethidium bromide assay, antiprolifertative activity, antioxidants, MTT assay, phenols, *Teucrium*

## Abstract

This study deals with total phenolic content, antiproliferative and proapoptotic activity of methanolic extracts from different *Teucrium* species and the effect on the prooxidant/antioxidant status in HCT-116 cells. The total phenolic content of the extracts was measured spectrophotometricaly and the obtained results ranged from 56.62 mg/g to 172.50 mg GA/g. The antiproliferative activity of methanolic extracts from different *Teucrium* species was determined using MTT cell viability assay, where IC_50_ value was used as a parameter for cytotoxicity. The type of cell death was explored by fluorescence microscopy using the acridin orange/ethidium bromide method. MTT assay showed that all extracts significantly reduced cell viability in a dose-dependent manner, with very low IC_50_ values. The highest content of phenolic compounds and the best cytotoxic activity on HCT-116 cells after 24 h of exposure was in *T. chamaedrys* extract, with IC_50_ values of 5.48 × 10^−9^ μg/mL. After 72 h, methanolic extract of *T. arduini* appeared to have the best cytotoxic activity on HCT-116, with IC_50_ values of 0.37 μg/mL. Treatments caused typical apoptotic morphological changes in HCT-116 cells and showed a high percentage of apoptotic cells. The results of the presented research indicate that some *Teucrium* extracts are a very rich source of phenols, which may directly contribute to high antiproliferative and proapoptotic activity.

## 1. Introduction

Throughout medical history, nature has long been shown to be an excellent and reliable source of new drugs, including anticancer agents. It is well established that plants have always been useful sources of antitumor or cancer prevention compounds [1,2]. Approximately more than 60% of currently used anticancer chemotherapeutic drugs are derived in one way or another from natural sources, including plants [3,4]. Large groups of different phenolic compounds from plants are important and essential anticancer agents [5,6]. In many cases, they are much more effective and do not have large unintended consequences compared with synthetic drugs. In fact, they are much studied in order to explore their further use in pharmacy and medicine in the prevention and treatment of cancer.

The genus *Teucrium* (Germander) belongs to the family Lamiaceae, within the subfamily Ajugoideae. In the flora of Europe, genus *Teucrium* has been divided into seven sections with 49 species. They are mostly perennial herbs, shrubs or subshrubs, while *T. botrys* is a herbaceous annual herb. The species of this genus are widespread on all continents of the world, and a very large number of species are present in the Mediterranean [7,8].

A large number of known medicinal species belonging to the genus *Teucrium* are used in folk medicine and pharmacy. The species of the genus *Teucrium* are very rich in phenolic compounds with very strong biological activity [9,10]. The most popular species of this genus are *T. chamaedrys*, *T. montanum* and *T. polium*, used in treatment of digestive and respiratory disorders, abscesses, gout and conjunctivitis, in the stimulation of fat and cellulite decomposition, and possess antiinflammatory, antioxidative, antimicrobial, antidiabetic and antihelmintic effects. However, their most significant therapeutic effect was the elimination of some problems in the digestive tract [11–13].

Recent studies suggest that plant extracts and isolated compounds from *Teucrium* species posses strong anticancer activity. Large numbers of studies show the importance of phenolic compounds in species of the genus *Teucrium* regarding the anticancer effects where distinctive properties were recognized. Extracts from some *Teucrium* species potentiate the cytotoxic and proapoptotic effects of anticancer drugs vincristine, vinblastine and doxorubicin against a panel of cancer cell lines [14]. The evaluation of the genus *Teucrium* as anticancer agents is unevenly enforced. There is no data on the anticancer activities and potential medicinal uses of some *Teucrium* species. Phenolics in plants have been reported to have a capacity to scavenge free radicals and strong antioxidant activity. The main antioxidant activity of plant extracts is largely due to their redox properties, which allow them to act as reducing agents, hydrogen donors and singlet oxygen quenchers [15].

Due to incomplete investigations of species in the genus *Teucrium*, there is no data in the literature concerning the comparative analysis of antiproliferative and antioxidant activity and quantitative analysis of phenolic compounds as active antitumor agents for known and important species of this genus. Therefore, the purpose of this study was to evaluate some *Teucrium* species as new potential natural sources of effective antiproliferative and antioxidant agent. *In vitro* antiproliferative activity and antioxidant properties on the HCT-116 human colon cancer cell line, as well as total phenolic contents of methanolic extracts from *T. montanum*, *T. chamaedrys*, *T. polium*, *T. arduini*, *T. botrys*, *T. scordium* subsp*. scordium* and *T. scordium* subsp*. scordioides* were investigated. To evaluate the antiproliferative activity we chose human colon cancer cell line, because we want to demonstrate the response of colon cancer cells on plants that have been used in the treatment of digestive disorders in traditional medicine. Also, colorectal cancer is a major cause of tumor-related morbidity and mortality worldwide [16].

## 2. Results and Discussion

Consistent epidemiological findings indicate that a diet with high consumption of antioxidant-rich fruits and vegetables significantly reduces the risk of many cancers. Plants and fruits could be effective agents for reducing cancer incidence and mortality. Plant-based diets could be used as preventive strategies to reduce the risk and inhibit or retard the development of colon cancer. The identification and development of such agents has become a major area of experimental cancer research and plant compounds may be explored for pharmaceutical application in the field of oncology [17,18].

In the present study, several plants belonging to the genus *Teucrium*, widely distributed in Serbia, were investigated regarding the concentration of phenolic compounds and possible anticancer properties on the HCT-116 cell line (antiproliferative effects as cells response to the *Teucrium* extracts). Large numbers of known medicinal species belonging to the genus *Teucrium* have been used in folk medicine and pharmacy. Species of the genus *Teucrium* are very rich in phenolic compounds with very strong biological activity. The total soluble phenolic content of the examined plant extracts, using the Folin-Ciocalteu method, are presented in Table 1. The content of total phenols in extracts, expressed as gallic acid equivalents (GA) per gram of dry extract, ranged between 56.62 and 186.02 mg GA/g. Very high concentrations of phenolic compounds were found in *T. scordium* subsp. *scordioides*, *T. scordium* subsp. *scordium*, *T. chamaedrys* and *T. montanum*; higher concentration was observed in *T. polium*, while *T. arduini* and *T. botrys* appeared to contain little lower concentration of phenolics.

The diversity of investigated *Teucrium* species resulted in varying concentrations of phenols in plant extracts. All investigated species of the genus *Teucrium* except *T. scordium* subsp. *scordioides*, *T. scordium* subsp. *scordium* xerophytes, were found to have xeromorphic structure of vegetative parts due to the arid environment. *T. scordium* with subsp. *scordioides* and subsp. *scordium* prefers humid habitat and has mesophytic characteristics. Among them, *T. botrys* is an annual plant, while others are perennial herbs [19]. Differences in life form are in accordance with the obtained concentrations of phenolic compounds.

The derivatives from several *Teucrium* species are dispensed for the treatment of obesity, hypercholesterolemia and diabetes, as well as for antiinflammatory, antimicrobial and anticancer properties [14]. There is data in literature about antiproliferative activity of *T. polium* on different cell lines [20], therapeutic promise in the treatment of human metastatic prostate cancer [21] and about *T. montanum* extract, which influenced cell growth in HeLa and MCF-7 cell lines [22]. There is no data in the literature about the antiproliferative activity of other *Teucrium* species.

In order to explore the antiproliferative activity of the methanolic extracts from different *Teucrium* species on the HCT-116 cell line, we conducted two *in vitro* experiments. We examined the cytotoxic effects of methanolic extracts (in concentration range from 50 to 1000 μg/mL) on the HCT-116 cell line using the MTT cell viability assay, as well as the proapototic effects we examined by AO/EB staining of treated HCT-116 cells.

**Figure 1:**
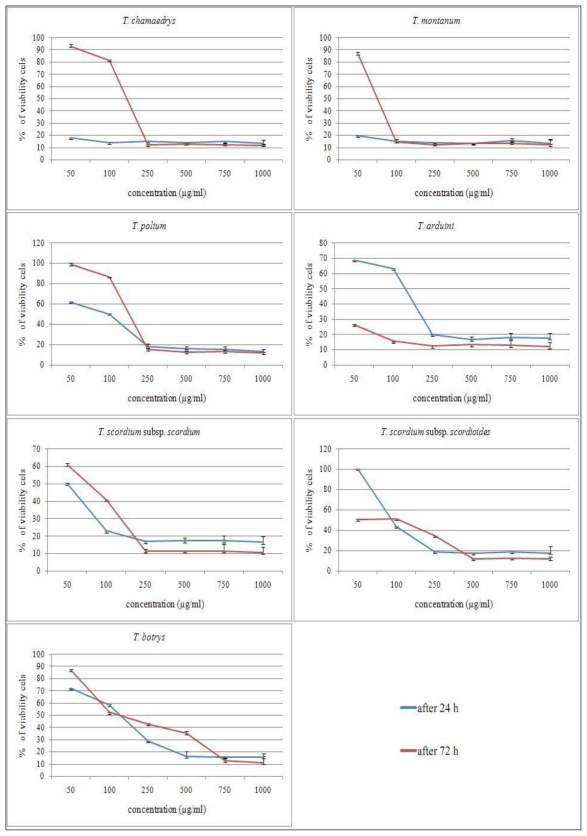
The dose-response curves of the effects of *Teucrium chamaedrys*, *T. montanum*, *T. polium*, *T. arduini*, *T. scordium* subsp. *scordium*, *T. scordium* subsp. *scordioides* and *T. botrys* on cell growth in HCT-116 cells. The cells were treated with various concentrations of drugs, after 24 h and 72 h of exposure. The antiproliferative effects were measured by MTT assay. Results were expressed as means ± SEM for three independent determinations.

A dose-dependent reduction of MTT activity (or color change from yellow to purple) was observed in extract-treated cells (Figure 1). The shape of dose-response curves indicates a significant inhibition of cell growth in dose-dependent manner in 24 h and 72 h of treatments. Cell growth was significantly lower (*p* < 0.05) if extract-treated cells were compared to control cells. The extracts exhibited higher effects after 72 h of exposure only at higher concentrations, but cytotoxic effects of lower concentrations were not higher after 72 h. The results indicate that cytotoxic effects of lower concentrations did not strengthen with increased exposure time, since the extracts have acute cytotoxic effect on the HCT-116 cell line and then, after longer exposure times, cells were recovered, except in the treatment with methanolic extract from *T. arduini*, which had a cytotoxic effect in a dose- and time-dependent manner. This means that low concentrations can kill cells immediately after treatment, but after longer times, they stimulate some proliferative effects in surviving cells (there is a high percent of viable cells 72 h after treatment), or cells have eventually adapted to the treatment. In addition, tested *Teucrium* species extracts could show time-dependent antioxidative and protective effects, the properties which we tested in the final part of this study.

Table 2 presents *in vitro* cytotoxic activity of the seven investigated methanolic extracts from different *Teucrium* species. The effects of extracts were expressed by IC_50_ values (inhibitory dose inhibited cell growth by 50%). The IC_50_ value was used as a parameter for cytotoxicity. Methanolic extract from *T. chamaedrys* had the maximum effect has after 24 h. The results demonstrated that three of seven investigated methanolic plant extracts had pronounced cytotoxic effects on the HCT-116 cell line (*T. chamaedrys—*5.48 × 10^−9^ μg/mL, after 24 h of exposure; *T. montanum—*1.08 × 10^−5^ μg/mL, after 24 h of exposure and *T. arduini—*0.37 μg/mL, after 72 h of exposure). The extracts from *T. scordium* subsp. *scordium* and *T. polium* exhibited a noteworthy cytotoxic effect after 24 h of exposure, as well as *T. scordium* subsp. *scordium*, *T. montanum* and *T. scordium* subsp. *scordioides* after 72 h of exposure. The other plant extracts may exhibit only a weak cytotoxic effect. According to the American National Cancer Institute (NCI), the criteria of cytotoxic activity for the crude extracts is IC_50_ < 30 μg/mL [23]. Considering the values of IC_50_, we can conclude that *T. chamaedrys*, *T. montanum*, *T. arduini* and *T. scordium* subsp. *scordium* have a strong antiproliferative effect on the HCT-116 cell line responding to the NCI criteria. Difference in cytotoxity of methanolic extracts from different species which belong to the genus *Teucrium* may be based on their chemical composition and difference between the effective components present in the extracts and their modes of action.

Phenolic compounds constitute one of the most numerous groups of plant metabolites. It has been found that phenols have a primary antioxidant activity, but this group of compounds showed a wide variety of biological functions related to the modulation of carcinogenesis. There are numerous data items regarding anticancer potential of natural phenolic compounds or extracts from different plant sources with growth-inhibitory effect on human cell lines, including HCT-116, in a dose-dependent manner with different sensitivity between cell lines [24–27]. Our data is in correspondence with the aforementioned research, because in comparison with phenol concentration values and antiproliferative activity of extracts, a notable correlation was observed, except for *T. scordium* subsp*. scordioides* where deviation was observed. Consequently, not only the concentration of phenolics but also the properties of these compounds contribute to the antiproliferative activities of different extracts.

The inhibitory effect of natural bioactive substances in carcinogenesis and tumor growth may be through two main mechanisms: modifying redox status and interference with basic cellular functions (cell cycle, apoptosis, inflammation, angiogenesis, invasion and metastasis [28]). Apoptosis has been reported to play an important role in the elimination of seriously damaged cells or tumor cells by chemopreventive or chemotherapeutic agents [29]. They are rapidly recognized by macrophages before cell lysis, and can then be removed without inducing inflammation. Therefore, apoptosis-inducing agents are expected to be ideal anticancer drugs.

Phenols from natural sources have been found to affect cancer cell growth by inducing apoptosis in many cell lines, including HCT-116 [30]. In order to determine whether the inhibition of cell proliferation by methanolic extracts from investigated plants were due to the induction of apoptosis, we used the acridine orange/ethidium bromide method. HCT-116 cells, treated with 250 μg/mL methanolic extracts of different *Teucrium* species, were stained with AO/EB and analyzed under a fluorescence microscope to calculate the percentage of viable, early and late apoptotic and necrotic cells.

The results obtained with AO/EB double staining are presented in Table 3. Compared with spontaneous apoptosis observed in control cells (early apoptotic 3.19%, 0% late apoptotic and 0% necrotic cells) HCT-116 treated with 250 μg/mL methanolic extracts from *T. chamaedrys*, *T. montanum*, *T. polium*, *T. arduini*, *T. scordium* subsp. *scordium*, *T. scordium* subsp. *scordioides* and *T. botrys* showed increased percentages of early apoptotic cells (the higest increase showed *T. chamaedrys—*55.65%), and late apoptotic cells (the highest increase showed *T. polium—*50.23%), and increased percentage of necrotic cells (the highest increase showed *T. botrys—*18.8%).

**Figure 2:**
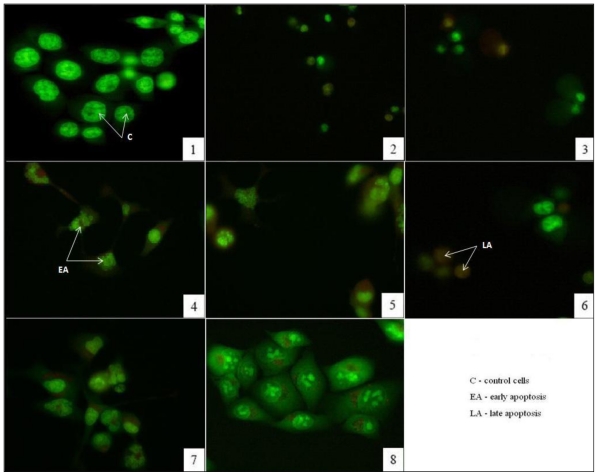
AO/EB staining of HCT-116 cells to detect apoptosis and necrosis induced by 250 μg/mL methanolic extracts from *T. chamaedrys* (2), *T. montanum* (3), *T. polium* (4), *T. arduini* (5), *T. scordium* subsp. *scordium* (6), *T. scordium* subsp. *scordioides* (7) and *T. botrys* (8) after 24 h of exposure. Untreated cells were observed as control cells (1). The images of cells were taken with a fluorescence microscope at 400×.

Proapoptotic activity of methanolic extracts from different *Teucrium* species regarding the morphological shape of cells was investigated by fluorescence microscopy. Florescence microscopic images clearly showed morphological changes such as cell shrinkage, membrane blebbing, chromatin condensation, nuclear fragmentation and formation of apoptotic bodies of treated cells (Figure 2). Therefore, the observations indicated that treatment with methanolic extracts rich in phenols induced apoptosis in the HCT-116 cell line. Results showed that *Teucrium* species used in the treatment of digestive disorders have strong antiproliferative and proapoptotic activities in the colon cancer cell line. Considering these important results, in order to complete all relevant data before possible therapeutic use, it is necessary to test effects of *Teucrium* species extracts on some normal cell line, as well as different colon cancer cell lines.

In order to explain the mechanism of antiproliferative and proapoptotic activities of *Teucrium* species extracts on the HCT-116 cell line, we followed prooxidative and antioxidative properties of investigated extracts. The data presented in Table 4 expressed the release of O_2_^−^ as nmol after 24 h and 72 h of incubation with seven methanolic plant extracts. *T. scordium* subsp. *scordioides* and *T. botrys*, at all concentrations (50, 250 and 500 μg/mL) produced significantly higher level of superoxide anion radical in HCT-116 cells compared to control after 24 h of treatment.

After 72 h of treatment, the highest level of superoxide anion radical production appeared, at concentration of 50 μg/mL, with *T. chamaedrys*, *T. arduini* and *T. botrys*; at concentration of 250 μg/mL with *T. chamaedrys* and *T. arduini* and at concentration of 500 μg/mL, with *T. arduini*, *T. scordium* subsp. *scordioides* and *T. botrys*, in comparison with control cells. We also compared the time of exposure of HCT-116 for 24 h and 72 h with various plant extracts and the results showed that 24 h treatment induces a higher level of superoxide anion radical when compared to 72 h treatment. Plant extracts of *T. scordium* subsp. *scordioides* and *T. botrys* induced the highest levels of superoxide anion radical.

Determination of the nitric oxide (NO) concentration demonstrated that all methanolic plant extracts were able to reduce the release of NO (Table 5) in comparison with control cells. Treatment with various plant extracts for 24 h induces a higher level of nitrites when compared to 72 h treatment. *T. chamaedrys*, *T. montanum*, *T. polium*, *T. arduini* and *T. scordium* subsp. *scordium* plant extracts significantly reduced the level of nitrites after 72 h of exposure, as low as 0 nmol/mL (detection barrier for Griess reaction). Our data is in correlation with one of antioxidant properties of phenolic compounds, since *T. chamaedrys*, *T. montanum*, *T. polium*, *T. arduini* and *T. scordium* subsp. *scordium* plant extracts significantly reduced the level of nitrites.

Previous reports demonstrated that many side effects of commonly used chemotherapy agents resulted from induction of oxidative stress, and that could be palliated by antioxidant food and plants uptake [31]. Some studies suggested that the antiproliferative effects of some polyphenol antioxidants on cancer cells are partially due to their prooxidant actions [32]. In our experiment, only methanolic extracts of *T. scordium* subsp. *scordioides* that has the highest phenol content induced the highest levels of superoxide anion radical after 72 h of treatment and maybe, at least in part, the source of its antiproliferative potential. On the other hand, due to their ability to scavenge and reduce the production of free radicals, and because they act as transition metal chelators, natural phenolic compounds may exert a major chemopreventive activity [28]. Our data showed that all methanolic extracts of *Teucrium* species had strong antioxidant properties after 72 h of treatment, since they reduce both levels of superoxide anion radical and nitrites. These antioxidative features may have some protective effects in surviving cells since there is a high percent of viable cells 72 h after treatment. For all these facts, plant could be used along with some stronger cytotoxic agents and chemotherapy agents (e.g., cisplatin), because they have strong immediate effects inducing apoptosis, but they lose effectiveness over time.

## 3. Experimental Section

### 3.1. Chemicals

Methanol, sodium hydrogen carbonate (NaHCO_3_), potassium hydroxide (KOH) and sodium nitrite (NaNO_3_) were purchased from “Zorka pharma”, Serbia. Gallic acid and rutin hydrate were obtained from Sigma Chemicals Co., St Louis, MO, USA. Folin-Ciocalteu phenol reagent, aluminium chloride (AlCl_3_) and *N*-(1-naphthyl)ethylenediamine were purchased from Fluka Chemie AG, Buchs, Switzerland. Dublecco’s Modified Eagle Medium (DMEM) was obtained from GIBCO, Invitrogen, USA. Fetal bovine serum (FBS) and trypsin-EDTA were from PAA (The cell culture company), Austria. Acridine orange was obtained from Acros organic, New Jersey, USA. Dimethyl sulfoxide (DMSO), nitro blue tetrazolium (NBT), ethidium bromide and 3-[4,5-dimethylthiazol-2-yl]-2,5- diphenyltetrazolium bromide (MTT) were obtained from SERVA, Germany and sulfanilic acid from MP Hemija, Serbia.

### 3.2. Plant Material

From June to September 2009 aerial flowering parts of *Teucrium* species were collected from natural populations in the region of Serbia and Montenegro. The voucher specimens of *T. arduini* L., *T. scordium* L. subsp. *scordium*, *T. scordium* L. subsp. *scordioides*, *T. botrys* L. *T. chamaedrys* L., *T. polium* L., *T. montanum* L., were confirmed and deposited in Herbarium at the Department of Biology and Ecology, Faculty of Science, University of Kragujevac. The collected plant material was air-dried in darkness at ambient temperature (20 °C). The dried plant material was cut up and stored in tightly sealed dark containers until needed.

### 3.3. Preparation of Plant Extracts

Prepared plant material (10 g) was transferred to dark-colored flasks and was soaked in 200 mL of methanol and stored at room temperature. After 24 h, the infusions were filtered through Whatman No. 1 filter paper and residue was re-extracted with equal volume of solvents. After 48 h, the process was repeated. Combined supernatants were evaporated to dryness under vacuum at 40 °C a using Rotary evaporator. The obtained extracts were kept in sterile sample tubes and stored in a refrigerator at 4 °C.

### 3.4. Determination of Total Phenolic Content in the Plant Extracts

The concentration of phenolics in the plant extracts was measured by using spectrophotometric method [33]. The methanol solution of the extract in concentration of 1 mg/mL was used in the analysis. The reaction mixture was prepared by mixing 0.5 mL of methanol solution of the extract, 2.5 mL of 10% Folin-Ciocalteu reagent dissolved in water and 2 mL of 7.5% NaHCO_3_. The blank was concomitantly prepared containing 0.5 mL of methanol, 2.5 mL of 10% Folin-Ciocalteu reagent dissolved in water and 2 mL of 7.5% of NaHCO_3_. The samples were thereafter incubated and thermostatically controlled at 45 °C for 45 min. The absorbance was determined using spectrophotometer at λ_max_ = 765 nm. The samples were prepared in triplicate for each analysis and the mean value of absorbance was obtained. The same procedure was repeated for the standard solution of gallic acid and the calibration line was construed. Based on the measured absorbance, the concentration of phenolics was read (mg/mL) from the calibration line; then the content of phenolics in the extracts was expressed in terms of gallic acid equivalent, (mg of Ga/g of extract).

### 3.5. Cell Preparation and Culturing

HCT-116 cell line was obtained from American Type Culture Collection. Cells were maintained in DMEM supplemented with 10% Fetal Bovine Serum, with 100 units/mL penicillin and 100 μg/mL streptomycin. Cells were cultured in a humidified atmosphere with 5% CO_2_ at 37 °C. Cells were grown in 75 cm^2^ culture bottles supplied with 15 mL DMEM, and after a few passages, cells were seeded in a 96-well plate. All studies were done with cells at 70 to 80% confluence.

### 3.6. Cell Viability Assay (MTT Assay)

HCT-116 cells were seeded in a 96-well plate (10,000 cells per well). After 24 h of cells incubation, the medium was replaced with 100 μL medium containing various doses of methanolic extracts at different concentrations (50, 100, 250, 500, 750 and 1000 μg/mL) for 24 h and 72 h. Untreated cells served as the control. After 24 h and 72 h of treatment the cell viability was determined by MTT assay [34]. The proliferation test is based on the color reaction of mitochondrial dehydrogenase in living cells by MTT. At the end of the treatment period, MTT (final concentration 5 mg/mL PBS) was added to each well, which was then incubated at 37 °C in 5% CO2 for 2–4 h. The colored crystals of produced formazan were dissolved in 150 μL DMSO. The absorbance was measured at 570 nm on Microplate Reader. Cell proliferation was calculated as the ratio of absorbance of treated group divided by the absorbance of control group, multiplied by 100 to give a percentage proliferation.

### 3.7. Fluorescence Microscopic Analysis of Cell Death

We used acridine orange/ethidium bromide (AO/EB) double staining assay [35]. Acridine orange is taken up by both viable and nonviable cells and emits green fluorescence if interrelated into double stranded nucleic acid (DNA) or red fluorescence if bound to single stranded nucleic acid (RNA). Ethidium bromide is taken up only by nonviable cells and emits red fluorescence by intercalation into DNA. We distinguished four types of cells according to the fluorescence emission and the morphological aspect of chromatin condensation in the stained nuclei. Viable cells have uniform bright green nuclei with organized structure. Early apoptotic cells (which still have intact membranes but have started to undergo DNA cleavage) have green nuclei, but perinuclear chromatin condensation is visible as bright green patches or fragments. Late apoptotic cells have orange to red nuclei with condensed or fragmented chromatin. Necrotic cells have a uniformly orange to red nuclei with condensed structure. The amount of 200 μL of dye mixture (100 μL/mg AO and 100 μL/mg EB in distilled water) was mixed with 2 mL cell suspension (30,000 cells/mL) in 6-well plate. The suspension was immediately examined and viewed under Nikon inverted fluorescence microscope (Ti-Eclipse) at 400× magnification. We observed untreated cells as controls and cells treated with methanolic extract of different *Teucrium* species in 250 μg/mL concentrations for 24 h of exposure. A minimum of 300 cells were counted in each sample.

### 3.8. Determination of Superoxide Anion Radical (NBT Assay)

The concentration of superoxide anion radical (O_2_^−^) in the sample was determined by spectrophotometric method [36], and is based on the reduction of nitroblue tetrazolium (NBT) to nitroblue-formazan in the presence of O_2_^−^. HCT-116 cells were seeded in triplicates in a 96-well plate (10,000 cells per well). After 24 h of cells incubation, the medium was replaced with 100 μL of medium containing various doses of methanolic extracts at different concentrations (50, 250 and 500 μg/mL) for 24 h and 72 h. Assay was performed by adding 100 μL of 5 mg/mL NBT to each well and then the cells were incubated for 3 h at 37 °C in 5% CO_2_. To quantify the formazan product, formazan was solubilized in 60 μL of 2 M KOH and DMSO and the resulting color reaction was measured spectrophotometrically on microplate reader at 570 nm (ELISA 2100C). The amount of NBT reduced was determined by the change in absorbance at 560 nm, based on molar extinction coefficient for monoformazan that is 15,000 M^−1^ cm^−1^ and the results were expressed as μmol/mL of cells.

### 3.9. Nitric Oxide (NO) Measurement

Experiments were performed at room temperature or at 37 °C in a warm room, as noted. Typically, a nitrite standard solution (100 mM) was serially diluted from 100 to 1.6 μM in triplicate in a 96-well, flat-bottomed, microtiter plate. All samples were seeded, also in triplicates in 96-well microtiter plate. Equal volumes of 0.1% (1 mg/mL) *N*-(1-naphthyl)ethylenediamine and 1% (10 mg/mL) sulfanilic acid (solution in 5% phosphoric acid) to form the Griess reagent were mixed together immediately prior to application to the plate. The spectrophotometric determination of nitrites–NO_2_^−^ (indicator of the nitric oxide-NO level) was performed by using the Griess method [37]. Briefly, the Griess reaction is a diazotization reaction in which the NO-derived nitrosating agent (e.g., N_2_O_3_), generated from the acid-catalyzed formation of nitrous acid from nitrite (or the interaction of NO with oxygen), reacts with sulfanilic acid to produce a diazonium ion that is then coupled to *N*-(1-napthyl)ethylenediamine to form a chromophoric azo product that absorbs strongly at 540 nm. The absorbance at 540 nm was measured by using a Micro Plate Reader (ELISA 2100C) following incubation (usually 5–10 min). The results were expressed in nmol nitrite/mL from a standard curve established in each test, constituted of known molar concentrations of nitrite.

### 3.10. Statistical Analysis

The data is expressed as means ± standard errors (SE). Biological activity was examined in three individual experiments, performed in triplicate for each dose. Statistical significance was determined using the Student’s *t*-test or the one-way ANOVA test for multiple comparisons. A *p* value <0.05 was considered as significant. The magnitude of correlation between variables was done using a SPSS (Chicago, IL) statistical software package (SPSS for Windows, version 17, 2008). The IC_50_ values were calculated from the dose curves by a computer program (CalcuSyn).

## 4. Conclusions

According to the results of investigations, *Teucrium* can be considered as a rich natural source of polyphenolic compounds. Our *in vitro* data indicated the inhibition of the HCT-116 cell line proliferation by methanolic extracts from different *Teucrium* species and induced apoptosis in the HCT-116 cell line. Among the investigated extracts, *T. chamaedrys*, *T. montanum*, *T. arduini* and *T. scordium* subsp. *scordium* have the strongest antiproliferative effects and could be considered as suitable candidates for further studies to find the effective anticancer components. Plant extracts can also act as antioxidants due to lower levels of nitrites, lower superoxide anion production and higher percentage of viable cells 72 h after treatment. Natural products from plants of this genus, as important medicines for a number of digestive diseases and disorders, represent the potential natural resources of effective substances in the treatment of digestive tract cancer.

## Figures and Tables

**Table 1:** Total phenolic contents 1 in the extracts in terms of gallic acid equivalent (mg of GA/g of extract).

Plant Species	Total Phenolic Content
*T. chamedrys*	172.50 ± 1.26
*T. montanum*	169.06 ± 0.75
*T. polium*	124.62 ± 1.05
*T. arduini*	90.39 ± 1.19
*T. s.* subsp. *scordium*	178.20 ± 1.11
*T. s.* subsp. *scordioides*	186.02 ± 0.91
*T. botrys*	56.62 ± 0.99

1 All values are mean ± SEM, *n* = 3.

**Table 2:** Growth inhibitory effects—IC_50_ values (μg/mL) of methanolic extracts of different *Teucrium* species—on HCT-116 cell line after 24 h and 72 h of exposure.

Plant Species	IC_50_ μg/mL	
	After 24 h	After 72 h
*T. chamaedrys*	5.48 × 10^−9^ ± 0.012	190.07 ± 3.28
*T. montanum*	1.08 × 10^−5^ ± 0.28	75.73 ± 2.71
*T. polium*	77.83 ± 0.41	253.39 ± 1.61
*T. arduini*	114.16 ± 0.26	0.37 ± 0.043
*T. s.* subsp. *scordium*	17.04 ± 0.47	59.02 ± 0.58
*T. s.* subsp. *scordioides*	143.46 ± 1.25	72.83 ± 1.56
*T. botrys*	116.38 ± 2.89	183.15 ± 3.15

All values are mean ± SEM, *n* = 3.

**Table 3:** Different values of viable, apoptotic and necrotic cells as percentage of all cells measured by AO/EB fluorescence staining, after treatment by 250 μg/mL of metanolic extracts from different *Teucrium* species. The percentages of cells were measured after 24 h of treatment.

Plant Species	Viable Cells	Early Apoptotic Cells	Late Apoptotic Cells	Necrotic Cells
control cells	96.35%	3.19%	-	-
*T. chamaedrys*	3.04%	55.65%	38.26%	3.04%
*T. montanum*	1.72%	54.02%	37.93%	6.32%
*T. polium*	0.91%	36.52%	50.23%	12.33%
*T. arduini*	1.29%	40.64%	47.09%	10.96%
*T. s.* subsp. *scordium*	0.72%	48.55%	44.20%	6.52%
*T. s.* subsp. *scordioides*	0.85%	40.17%	40.17%	18.80%
*T. botrys*	52.49%	47.51%	-	-

**Table 4:** Effect of methanolic extracts from different *Teucrium* species, on HCT-116 cell line after 24 h and 72 h of exposure, on superoxide anion radical (O_2_^−^) production expressed as μmol/mL of cells.

Plant Species	0 μg/mL	50 μg/mL	250 μg/mL	500 μg/mL

	After 24 h			
*T. chamaedrys*	38.28 ± 0.46	40.73 ± 0.24 *#	39.22 ± 0.38 *#	39.42 ± 0.20 *#
*T. montanum*	38.28 ± 0.46	40.63 ± 0.14 *#	38.73 ± 0.71 #	39.48 ± 0.12 *#
*T. polium*	38.28 ± 0.46	40.24 ± 0.36 *#	38.73 ± 0.19 #	39.58 ± 0.33 *#
*T. arduini*	38.28 ± 0.46	40.43 ± 0.18 *#	39.56 ± 0.20 *#	39.37 ± 0.19 *#
*T. s.* subsp. *scordium*	38.28 ± 0.46	41.76 ± 0.87 *#	39.8 ± 0.19 *#	38.94 ± 0.09 #
*T. s.* subsp. *scordioides*	38.28 ± 0.46	40.63 ± 0.09 *#	40.21 ± 0.19 *#	40.13 ± 0.16 *#
*T. botrys*	38.28 ± 0.46	41.55 ± 0.16 *#	40.6 ± 0.22 *#	39.49 ± 0.20 *#

	**After 72 h**			

*T. chamaedrys*	5.35 ± 0.24	5.76 ± 0.26	5.91 ± 0.16 *	5.15 ± 0.21
*T. montanum*	5.35 ± 0.24	5.39 ± 0.21	5.48 ± 0.15	5.19 ± 0.16
*T. polium*	5.35 ± 0.24	5.43 ± 0.22	5.06 ± 0.13	5.07 ± 0.21
*T. arduini*	5.35 ± 0.24	5.78 ± 0.16	5.69 ± 0.18	5.82 ± 0.56
*T. s.* subsp. *scordium*	5.35 ± 0.24	5.58 ± 0.19	5.84 ± 0.23 *	5.88 ± 0.45
*T. s.* subsp. *scordioides*	5.35 ± 0.24	5.47 ± 0.21	5.21 ± 0.27	6.24 ± 0.21 *
*T. botrys*	5.35 ± 0.24	5.96 ± 0.15 *	5.35 ± 0.18	5.89 ± 0.34

All values are mean ± SEM, *n* = 3.

* *p* < 0.05 as compared with control and

# *p* < 0.05 after 24 h and 72 h of treatment.

**Table 5:** Effect of methanolic extracts from different *Teucrium* species, on HCT-116 cell line after 24 h and 72 h of exposure, on the nitrite (NO_2_^−^) production expressed as nmol/mL of cells.

Plant species	0 μg/mL	50 μg/mL	250 μg/mL	500 μg/mL

	After 24 h			
*T. chamaedrys*	12.36 ± 0.99	3.3 ± 0.68 *#	1.77 ± 0.21 *	4.15 ± 0.43 *#
*T. montanum*	12.36 ± 0.99	11.06 ± 3.94 #	1.46 ± 0.55 *#	1.18 ± 0.42 *
*T. polium*	12.36 ± 0.99	5.78 ± 1.55 *	3.24 ± 1.01 *#	1.21 ± 0.52 *
*T. arduini*	12.36 ± 0.99	15.44 ± 4.42 #	3.79 ± 1.4 *	9.91 ± 3.46
*T. s.* subsp. *scordium*	12.36 ± 0.99	2.2 ± 0.31 *	2.32 ± 0.89 *	2.09 ± 0.19 *#
*T. s.* subsp. *scordioides*	12.36 ± 0.99	14.29 ± 6.66	7.34 ± 2.76	11.17 ± 5.16
*T. botrys*	12.36 ± 0.99	10.83 ± 2.83 *#	4.98 ± 1.69	2.57 ± 0.37 *

	**After 72 h**			

*T. chamaedrys*	3.23 ± 0.73	0.36 ± 0.36 *	1.09 ± 0.38 *	0.98 ± 0.32 *
*T. montanum*	3.23 ± 0.73	0.01 ± 0.01 *	0.08 ± 0.08 *	0.81 ± 0.38 *
*T. polium*	3.23 ± 0.73	1.56 ± 1.27	0.01 ± 0.01 *	0.23 ± 0.23 *
*T. arduini*	3.23 ± 0.73	0.3 ± 0.3 *	0.15 ± 0.15 *	2.43 ± 0.77
*T. s.* subsp. *scordium*	3.23 ± 0.73	2.08 ± 1.63	1.12 ± 0.53 *	0.39 ± 0.39 *
*T. s.* subsp. *scordioides*	3.23 ± 0.73	3.5 ± 0.76	1.59 ± 0.56	3.59 ± 0.17
*T. botrys*	3.23 ± 0.73	0.59 ± 0.49 *	2.4 ± 0.81	1.34 ± 0.59 *

All values are mean ± SEM, *n* = 3;

* *p* < 0.05 as compared with control and

# *p* < 0.05 after 24 h and 72 h of treatment.
